# Perspectives given by structural connectivity bridge the gap between structure and function

**DOI:** 10.1007/s00429-020-02080-z

**Published:** 2020-05-15

**Authors:** Hiromasa Takemura, Michel Thiebaut de Schotten

**Affiliations:** 1 Center for Information and Neural Networks (CiNet), National Institute of Information and Communications Technology, and Osaka University, Suita-shi, Japan; 2 Graduate School of Frontier Biosciences, Osaka University, Suita-shi, Japan; 3 Brain Connectivity and Behaviour Laboratory, Sorbonne Universities, Paris, France; 4 Groupe D’Imagerie Neurofonctionnelle, Institut Des Maladies Neurodégénératives-UMR 5293, CNRS, CEA University of Bordeaux, Bordeaux, France

“Afferent, efferent and intrinsic connections, as well as cell types and their properties, are the structural basis of a brain region's function” (Zilles and Amunts 2015). Over the last several decades, neuroscience has made enormous progress in understanding brain-function mechanisms at different spatial scales, ranging from the single-neuron level to macroscale cortical maps. While some reports demonstrate that a function can be localised into a specific area (localisationism), a collection of neuroscience studies also indicate that functions are mediated through the interaction of multiple brain areas. We will argue that extreme localisationism thinking has lost perspective. While areas can be sensitive to specific functions, they are not independently processing the information. For instance, reading this text requires the involvement of a system of interconnected brain areas analysing visual words and phonological and lexical information (Wandell et al. 2012). Hence, there is a pressing need to understand ‘structural connectivity’, which is a term generally referring to anatomical connections between brain areas. Structural connectivity is essential in understanding the circuitry supporting the interaction between brain areas and in bridging anatomy with function.

Despite many connectomics projects (Bakker et al. 2012; Burns et al. 2013; Van Essen et al. 2013; Zingg et al. 2014; Oh et al. 2014; Majka et al. 2020), a comprehensive understanding of structural connectivity of the human brain is still missing. While several methods for studying structural connectivity have been developed, they all present a trade-off between advantages and limitations. For instance, higher spatial resolution comes with a smaller field of view, while other methods, typically diffusion magnetic resonance imaging (dMRI), have a lower resolution but cover the whole brain. Similarly, some methods are only applicable to ex vivo animal brains, while other methods are available for living human brains. A consensus on the whole picture of the structural connectivity is challenged by these limitations (Rushmore et al. 2020). ‘Structural connectivity’ is also a multidimensional concept that is far from the simplified notion of ‘connected’ or ‘not connected’. At a cellular/molecular level, the type of synapses will impact the underlying neural circuitry with quite different functional implications (Bargmann and Marder 2013). At a macroscopic level, there is increasing evidence from dMRI studies showing that differences in white matter bundles can explain behavioural diversity among human individuals (Catani et al. 2007; Thiebaut de Schotten et al. 2011; Huber et al. 2018; Oishi et al. 2018), as well as impact precision medicine (Forkel et al. 2014, 2020; Takemura et al. 2019; Forkel and Thiebaut de Schotten 2020).

This new Brain Structure and Function special issue entitled ‘Structural connectivity of the cerebral cortex’ aims at providing the reader with a comprehensive understanding of the organisation of the brain’s structural connectivity, based on different methods (Lanciego and Wouterlood 2020; Blazquez Freches et al. 2020; Huang et al. 2020; Oishi et al. 2020; Kaneko et al. 2020; Woodward et al. 2020). The special issue also clarifies the multidimensional concept of structural connectivity by collecting articles that investigate how its variations at different scales affect brain functions (Rockland 2020; Gamberini et al. 2020; Andre et al. 2020; Ioannucci et al. 2020; Rooks et al. 2020). Finally, we invited investigators developing cutting-edge clinical applications of structural connectivity to provide an insight into how measuring this connectivity in the human brain can benefit society (Vanderweyen et al. 2020; David et al. 2020).

Typically, structural connectivity is derived from chemical tracers applied to non-human primate brains (Schmahmann and Pandya 2006). Since this original work, new types of tracers appeared with different properties that render interpretation difficult for non-experts. Furthermore, making raw tracer data machine-readable for quantitative analyses remains a challenge. Lanciego and Wouterlood (2020) (this issue) reviewed a wide range of neuroanatomical tract-tracing methods, from classical to modern, and examined their advantages and disadvantages. Woodward et al. (2020) (this issue) also proposed a new tracer processing pipeline in marmoset brains. They succeeded at quantifying anterograde tracer signals by using the latest artificial intelligence algorithms. They included three-dimensional reconstructions of tracer and histological data, as well as registration to a standard brain space as an MRI dataset, which bridges the gap between tracer data and the aforementioned dMRI data.

Other studies in this special issue demonstrate that direct dMRI data from the living human brain can show its connectional organisation. Accordingly, Blazquez Freches et al. (2020) (this issue) used dMRI-based tractography to identify the essential principles of structural connectivity in the human temporal cortex. They identified three connectivity gradients that displayed, within the temporal cortex, distinct connections that were supported by different white matter tracts and showed specific relationships to functions. The brain can also be divided into subregions (i.e. parcellation) according to the areas they preferentially connect. These methods are often tricky to implement; therefore, Reuter et al. (2020) (this issue) provided open-source software that makes the connectivity-based parcellation method broadly accessible to the neuroscience community. dMRI can also estimate  further spatial details of the white matter. For instance, Huang et al. (2020) (this issue) proposed a framework to determine the diameter of white matter axons in the living human brain. Oishi et al. (2020) (this issue) performed dMRI acquisitions on an ex vivo human brain with a high spatial resolution to characterise the white matter organisation in the subthalamic area. Finally, Kaneko et al. (2020) (this issue) used high spatial resolution dMRI data from the common marmoset to investigate occipital white matter tracts and clarify the similarities and differences in the visual system fibre tracts across primate species. Hence, despite some considerable advances, these studies make clear that a comprehensive understanding of the white matter anatomy, particularly in humans, is still missing and crucially needed.

Knowledge derived from the anatomical organisation of white matter goes well beyond anatomy, as it contributes to the understanding of functional circuitry and cortical dynamics (Rockland 2020, this issue). For instance, single-axon analyses have revealed essential organisation principles of thalamocortical or cortico-cortical connections, such as laminar organisation and the existence of axon collaterals and intrinsic/extrinsic connections. Specifically, differences in laminar specificity between feedforward and feedback connections, together with collaterals within and across areas, provide essential insights into the recurrent nature of cortical visual processing. Hence, the discovery of white matter principles offers critical information to interpret the neural dynamics of intra-areal communications, as well as the primary mechanisms supporting functions and pathologies.

In line with this statement, the review from Gamberini et al. (2020) (this issue) provides a comprehensive overview of the organisation of the superior parietal lobule (SPL), which combines cytoarchitecture, structural connectivity and electrophysiology together with functional MRI. The study indicates that while macaque SPL has often been considered to be entirely contained within Brodmann’s area 5, it can be divided into several regions with distinct connections to somatosensory, motor, visual and frontal cortices. Importantly, functional differences among these areas may be associated with anatomical connectivity differences, such as the degree of neuronal modulation by visual inputs or involvement in the control of limb movement. Another example of function being tightly linked with structural connections includes the relationship between the microstructural properties of white matter tracts in the limbic system and the sub-clinical diversity of internal and external behaviour among children and adolescents (Andre et al. 2020) (this issue). Similarly, the lateralisation of the limbic tracts shown in Ioannucci et al. (2020) (this issue) is related to the asymmetrical facial expression of happiness and sadness. Finally, the connectivity matrices derived from dMRI data demonstrate some fair differences according to everyday decision-making capacity in older age (Rooks et al. 2020) (this issue). These examples highlight the importance of white matter connectivity to the functional organisation of the brain and the differences in behaviour observed.

A better understanding of structural connectivity in humans can also impact our society. David et al. (2020) (this issue) discovered a relationship between the tissue property of specific fibre tracts and aggressive behaviours after military deployment, which may help with the early identification of soldiers who need an intervention procedure. Vanderweyen et al. (2020) (this issue) reviewed the role of dMRI in the optimisation of neurosurgical resection strategy and outcome. These papers provide a perspective on promising future applications to address contemporary problems outside academia.

While this special issue covered a wide range of topics and methods, it does not include other promising approaches including polarised light imaging (Axer et al. 2011; Wang et al. 2018; Caspers and Axer 2019), tissue clearing methods (Hama et al. 2011; Chung and Deisseroth 2013), and expansion microscopy (Wassie et al. 2019). As these measurements rely on different principles and concepts, additional studies comparing these methods with ones presented here will be essential to link the different spatial scales and provide a comprehensive understanding of structural connectivity. Other avenues, such as how neural dynamics are related to the underlying structural connectivity, remain utterly open for future generations to explore. We wish that collaborative efforts among neuroanatomists with different disciplines will help address these difficult remaining issues in the next several decades.
